# Rapid Eye Movement Sleep Deprivation Induces Neuronal Apoptosis by Noradrenaline Acting on Alpha1 Adrenoceptor and by Triggering Mitochondrial Intrinsic Pathway

**DOI:** 10.3389/fneur.2016.00025

**Published:** 2016-03-07

**Authors:** Bindu I. Somarajan, Mudasir A. Khanday, Birendra N. Mallick

**Affiliations:** ^1^School of Life Sciences, Jawaharlal Nehru University, New Delhi, India

**Keywords:** BAD, caspases, cytochrome *c*, locus coeruleus, neuronal degeneration

## Abstract

Many neurodegenerative disorders are associated with rapid eye movement sleep (REMS) loss; however, the mechanism was unknown. As REMS loss elevates noradrenaline (NA) level in the brain as well as induces neuronal apoptosis and degeneration, in this study, we have delineated the intracellular molecular pathway involved in REMS deprivation (REMSD)-associated NA-induced neuronal apoptosis. Rats were REMS deprived for 6 days by the classical flower pot method; suitable controls were conducted and the effects on apoptosis markers evaluated. Further, the role of NA was studied by one, intraperitoneal (i.p.) injection of NA-ergic alpha1 adrenoceptor antagonist prazosin (PRZ) and two, by downregulation of NA synthesis in locus coeruleus (LC) neurons by local microinjection of tyrosine hydroxylase siRNA (TH-siRNA). Immunoblot estimates showed that the expressions of proapoptotic proteins viz. Bcl2-associated death promoter protein, apoptotic protease activating factor-1 (Apaf-1), cytochrome *c*, caspase9, caspase3 were elevated in the REMS-deprived rat brains, while caspase8 level remained unaffected; PRZ treatment did not allow elevation of these proapoptotic factors. Further, REMSD increased cytochrome *c* expression, which was prevented if the NA synthesis from the LC neurons was blocked by microinjection of TH-siRNA *in vivo* into the LC during REMSD in freely moving normal rats. Mitochondrial damage was re-confirmed by transmission electron microscopy, which showed distinctly swollen mitochondria with disintegrated cristae, chromosomal condensation, and clumping along the nuclear membrane, and all these changes were prevented in PRZ-treated rats. Combining findings of this study along with earlier reports, we propose that upon REMSD NA level increases in the brain as the LC, NA-ergic REM-OFF neurons do not cease firing and TH is upregulated in those neurons. This elevated NA acting on alpha1 adrenoceptors damages mitochondria causing release of cytochrome *c* to activate intrinsic pathway for inducing neuronal apoptosis in REMS-deprived rat brain.

## Introduction

Our understanding about the causes and mechanism of action of the neurodegenerative diseases is still poor. For a comprehensive understanding, a thorough knowledge of their cause and effect as well as their cellular and molecular mechanism(s) of occurrence, preferably *in vivo* was needed. Many such diseases including Alzheimer’s, Parkinson’s, etc., are related to aging, whose population is rising exponentially throughout the world. Aging-associated disorders so to say are inevitable; however, their expressions depend on predisposition and susceptibility, which are modulated by changes in one or more fundamental physiological processes. One of the common fundamental physiological processes affected in most such disorders is changes in sleep–waking, which is also affected by lifestyle and *vice versa*. Sleep has been divided into rapid eye movement sleep (REMS) and non-REMS, and as a common factor, at least the former is affected in most of the psychosomatic and neurodegenerative disorders. We have shown earlier that experimental REMS deprivation (REMSD) affects neuronal cytomorphology and structural protein leading to neuronal apoptosis and loss, and these effects were mediated by REMSD-associated elevation in the level of noradrenaline (NA) in the brain (1–3). Our contention gets supported by a recent report in humans that gray matter volume is reduced upon sleep disturbance (4). The NA level in the brain is affected after REMSD (5, 6) and elevated NA has been reported to affect many of the REMSD (including total sleep loss)-associated effects, for example, thermoregulation (7), feeding (8), irritability and excitability (9), learning and memory (10), and psychosomatic disorders (11–13). A pathological condition characterized by death of neurons in the dorsal raphe nucleus and locus coeruleus (LC) in Alzheimer’s and Parkinson’s disease is also observed where REMS loss is a common phenomenon. Our previous study showed changes in BAX and Bcl2 proteins in REMSD rat brain (1–3), indicating a possibility of involvement of mitochondria-associated proteins in REMSD-induced apoptosis. As there was a need to understand the molecular mechanism of REMSD-associated neurodegeneration, in this study, we have investigated the intracellular mechanism of REMSD-associated NA-induced neuronal apoptosis in the rat brain. We have observed that REMSD-associated neuronal apoptosis followed mitochondrial intrinsic pathway by releasing cytochrome *c* and by affecting the Akt survival pathway. Further, the role of NA in inducing such apoptosis was confirmed *in vivo*; one, by blocking adrenoceptors and two, by downregulation of NA synthesis in REMS-deprived rat brains.

## Materials and Methods

### Animals and REMS Deprivation

A total of 55 adult male Wistar rats (250–280 g) maintained under 12:12 light:dark cycle at controlled temperature (25 ± 1°C) in standard laboratory conditions having free access to *ad libitum* food and water were used in this study. All experimental procedures were approved by the Institutional Animal Ethics Committee of the Jawaharlal Nehru University, New Delhi, India. Free moving control (FMC) rats (*N* = 5) were maintained in their normal dry home cages. The rats (*N* = 5) were REMS deprived by the classical flower pot method for 6 days (14, 15). In brief, the experimental rats were maintained on 6.5-cm diameter platform surrounded by water. In order to exclude the effects of non-specific confounding factors, a separate group of rats (*N* = 5) were maintained on larger platform of 12.5-cm diameter, the large platform control (LPC) group. The recovery (REC) group included animals (*N* = 5) deprived of REMS for 6 days and then allowed to live in normal cages for 3 days to recover from lost REMS. In another set (*N* = 5), 2 mg/kg/day prazosin (PRZ) was i.p. injected between 10:00 and 11:00 a.m. into the rats on each day of the last 4 days (i.e., third to sixth day) of 6-day REMSD schedule as before (3). At the end of the experiments, the rats were sacrificed between 10:00 and 11:00 a.m. by cervical dislocation, and the brains were taken out for further studies.

### Stereotaxic Surgery

Under isofluorane- (Baxter, Baxter Healthcare Corporation of Puerto Rico, USA) induced surgical anesthesia and aseptic condition, rats were stereotaxically implanted with stainless steel guide cannulae bilaterally targeting the LC (AP = −9.68, *L* = 1.3, and *H* = 7.4) (16, 17). The guide cannulae were advanced into the brain so that their tips in the brain reached 1 mm above the target and fixed to the skull with dental acrylic cement. About a week was allowed with adequate post-operative care to recover the rats from postsurgical trauma before experiments were conducted on them (18).

### REMSD and Microinjection of TH-siRNA into LC

**Figure 1:**
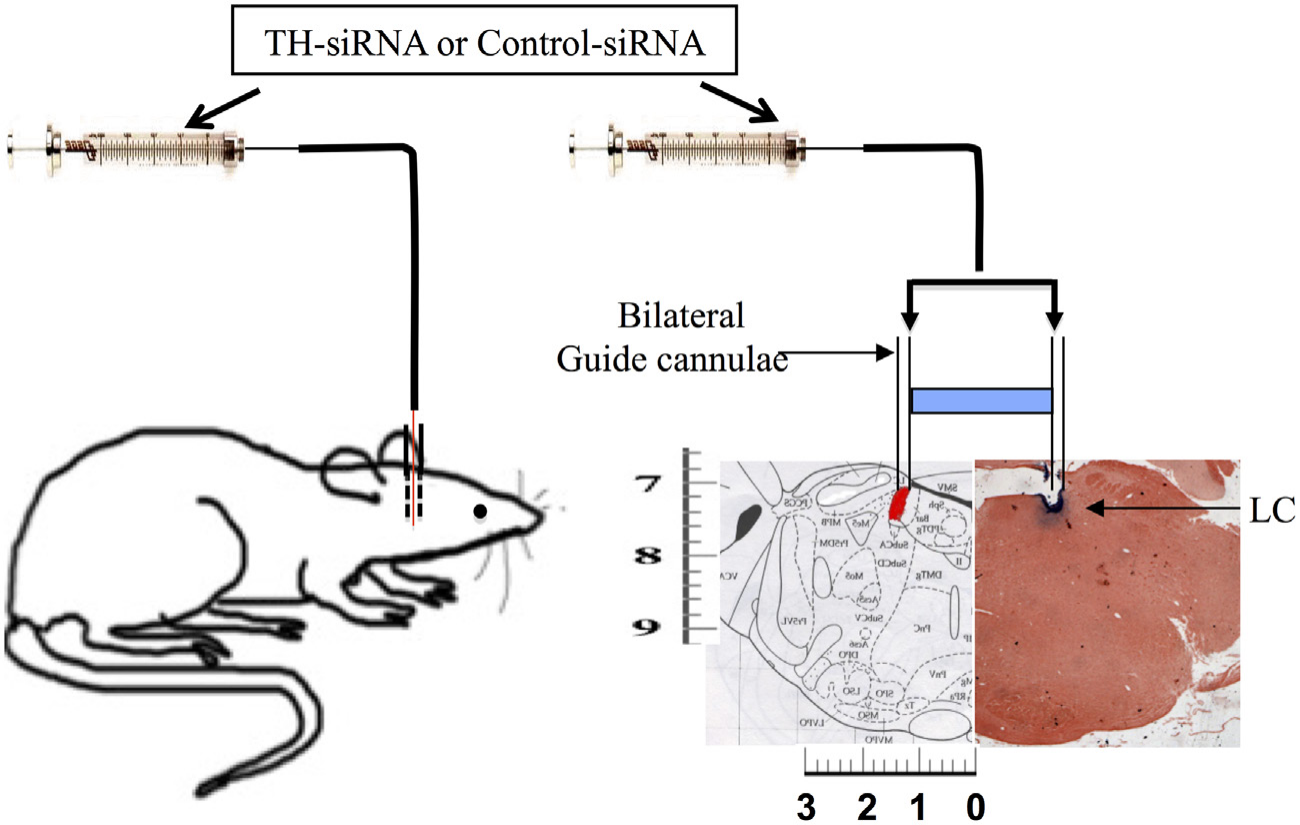
**Schematic representation of microinjection of TH-siRNA and control siRNA *in vivo* bilaterally into the LC of freely moving normally behaving surgically prepared chronic rats**.

Morphological changes associated with neuronal apoptosis could be observed only after 144 h REMSD; however, the molecular signaling cascade of apoptosis may commence with initiation of increased NA level during REMSD. Therefore, we conducted the tyrosine hydroxylase siRNA (TH-siRNA) experiment for 96 h so that distress to the experimental rats was minimized. After recovery from surgical trauma, the chronically prepared rats were divided into three groups of five rats each: FMC which did not receive any microinjection (treatment), 96 h REMS deprived which received TH-siRNA bilaterally into the LC, and another control group of 96 h REMS-deprived rats which received control siRNA (Ambion, USA) bilaterally into the LC. The sense-5′CUGUGAAGUUUGACCCGUAtt-3′ and antisense-5′UACGGGUCAAACUUCACAgg-3′ TH-siRNA (Sigma, USA) were incubated with N-TER (Sigma, USA) for 30 min before injection. During the REMSD period for two consecutive days, 0.5 μl containing 0.01 nmol of either TH-siRNA or control siRNA was bilaterally microinjected into the LC (Figure 1) at the end of 24 and 48 h of REMSD and deprivation continued for a total of 96 h. The microinjections into the LC in the chronically prepared freely moving otherwise normally behaving rats were done as described earlier (16, 17). Briefly, it was done using a 33G stainless steel injector smoothly passing through the guide cannulae already implanted into the rat brain. The injector had a stopper arrangement so that when introduced through the guide cannulae, the tip of the injector projected 1 mm out of the guide cannulae inside the brain to reach the target, the LC. A 2-μl Hamilton syringe was connected to the injector with approximately 10 cm polyethylene tubing containing the solution to be injected. The rat was held briefly for a period of about 5–6 min during microinjection on each side LC; there was a gap of about 5 min between injections into each side. After completion of experiment, the rats were sacrificed between 10:00 and 11:00 a.m. by cervical dislocation, and the brains were taken out for further analysis. As the study demanded, the rat brains to be homogenized for other protein assays, it was a technical limitation to confirm the injection site histologically in the brains of the same rat. As an alternative, we evaluated TH band intensity by Western blot in samples from brain homogenate in experimental and control rat brains. As LC is the primary site for NA-ergic neurons in the brain and all other conditions remaining identical (except microinjection of TH-siRNA and control siRNA into the LC in control and experimental rats), the TH-band density in the Western blot reflected the TH expressions in the LC neurons. This was supported in pilot studies where the microinjection site was histologically confirmed in the LC (Figure 1).

### Electron Microscopy

Experimental rats were intracardially perfused, and approximately 1 mm thick brain tissue blocks having LC, as per the atlas of Paxinos and Watson (19), were cut as reported earlier (1) and fixed for 24 h in Karnowsky fixative (2% paraformaldehyde and 2% glutaraldehyde solution; pH 7.2). The blocks were then transferred into PBS and subsequently processed for transmission electron microscopy (TEM) at the Advanced Instrumentation Research Facility of Jawaharlal Nehru University. The tissues were post-fixed in 1% osmium tetroxide (Sigma, USA) for 2 h at 4°C, dehydrated in grades of ethanol, and cleared in toluene before preparing araldite blocks for ultra-thin sectioning. The sections were collected on metal grids and stained using uranyl acetate and lead citrate. After washing in distilled water, the grids were stored in airtight petri plates for viewing under TEM. TEM experiments included *N* = 15, three rats in each group of FMC, LPC, REMSD, REC, and PRZ.

### Antibody and Reagents

Rabbit polyclonal primary antibodies anti-Apaf-1 (ab2001), anti-Bcl2-associated death promoter (BAD) (ab90435), anti-Caspase3 (ab47131), anti-Caspase9 (ab2324), and anti-pAkt (ab66138) were commercially obtained from Abcam (Cambridge, UK). Anti-TH (AB152), anti-caspase8 (AB1879), and anti-Cytochrome *c* (3025-100) were purchased from Millipore, CA, USA and Biovision, CA, USA, respectively. Horseradish peroxidase (HRP)-conjugated goat anti-rabbit secondary antibodies (sc-3837) were purchased from Santa Cruz Biotechnology, USA. Monoclonal mouse anti-tubulin antibody (05-829) from Millipore, USA was used for loading control. PRZ was procured from Sigma-Aldrich, St. Louis, MO, USA.

### Immunoblotting

At the end of the experiment, the control and the experimental rats were decapitated after spinal dislocation. The brains (including the cerebellum and brainstem) were quickly removed individually and homogenized separately in 10-ml ice cold homogenization buffer containing 0.32M sucrose, 12 mM Tris (pH 7.4), and 1 mM EDTA. The brain homogenate was centrifuged at 3000 × *g* (6000 rpm) for 5 min at 4°C to remove the larger debris. The supernatant was centrifuged at 11,000 × *g* (12,000 rpm) for 20 min at 4°C. After centrifugation, 2 ml of the supernatant was carefully removed for Western blotting; protein concentration was determined by Lowry’s method (20). For Western blotting, equal amount of proteins were loaded in separate wells in 8 or 10% polyacrylamide gels and allowed to run electrophoretically using standard SDS-PAGE protocol (21, 22). Following electrophoresis, the separated proteins on the gels were electrotransferred on to 0.45-μm nitrocellulose membrane (MDI, India) using a Trans-Blot SD (Bio-Rad, USA). After blocking the membranes with 3% BSA in Tris-buffered saline (TBS) for 3 h at room temperature, the membranes were incubated overnight at 4°C with primary antibodies. The membranes were then washed with TBS containing 0.1% Tween-20 (TBS-T) and probed with appropriate HRP-conjugated secondary goat anti-rabbit antibody for 1.5 h at room temperature. After secondary antibody incubation, the membranes were washed three times with TBS-T followed by with TBS. Protein expressions were visualized by enhanced chemiluminescence method using Clarity™ (Bio-Rad, USA). The band densities (after background subtraction) on the film were estimated using alpha image software (Alpha Innotech, USA). As the α-tubulin band intensities among the controls and experimental rat brain samples were comparable, the relative changes in the intensity of each of the protein bands of interest under various conditions (LPC, REMSD, REC, PRZ, control-siRNA and TH-siRNA) in every gel were estimated against respective FMC band intensity taken as 100% for normalization.

### Statistics

Data were collected from five independent sets of experiments; each set consisted of one rat from each group and the values have been expressed as mean ± SEM. The differences in the mean intensities of the protein bands of interest between experimental and control rat brains were statistically compared with that of the FMC using Sigma Stat Statistical Software version 12. As one-way ANOVA did not show any significant intra-group variation, the data from five rats within each group were pooled and inter-group significance levels evaluated applying Student–Newman–Keuls *post hoc* test; at least *p* < 0.05 was considered statistically significant.

## Results

### Bcl-2-Associated Death Promoter Expression

**Figure 2:**
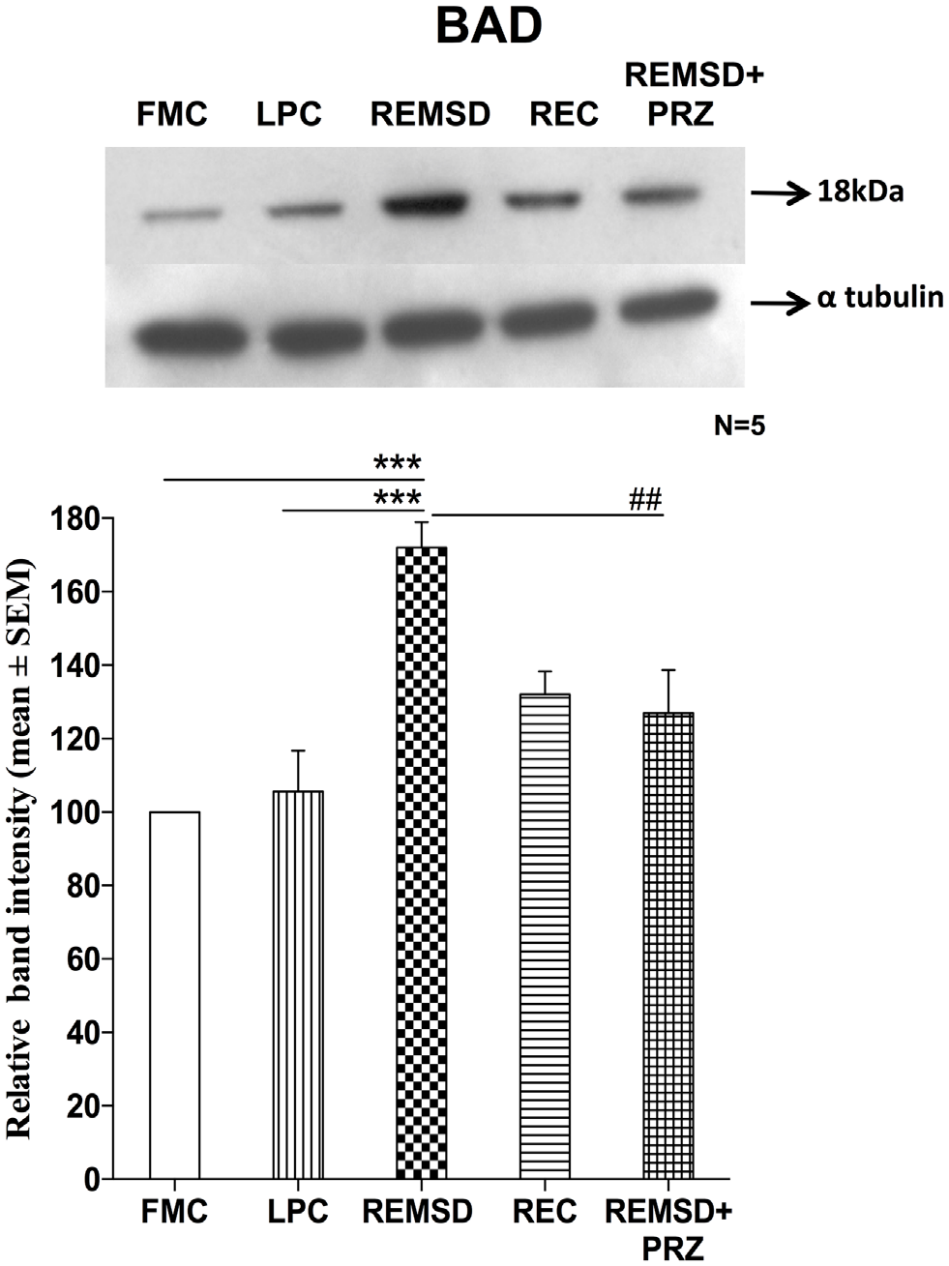
**BAD expression in the brain homogenate of rats under different conditions**. Upper panel shows a representative Western blot of BAD expressions in the brain of rats exposed to various control and experimental (REMSD) conditions. Histogram in the lower panel shows percent changes in the mean (±SEM) band densities of the blots as compared to FMC taken as 100% in five sets of experiments. Abbreviations are as in the text. Significance levels are between the treatments of connecting horizontal bars; ***p < 0.001 and ^###^*p* < 0.001, * as compared to FMC and ^#^ as compared to REMSD.

Rapid eye movement sleep deprivation significantly increased BAD expression in the whole brain homogenate as compared to FMC [*F*_(1,8)_ = 58.31, *p* < 0.001] and REC [*F*_(1,8)_ = 32.03, *p* < 0.001] rat brain samples. The BAD expressions in LPC were unaffected and remained comparable to FMC [*F*_(1,8)_ = 0.20, *p* < 0.66]. PRZ prevented the increase in BAD expression in REMSD rat brain [*F*_(1,8)_ = 16.03, *p* < 0.004]; however, the levels remained higher [*F*_(1,8)_ = 5.00, *p* < 0.05] than that of the sample from FMC rat brains (Figure 2).

### Cytochrome *c* Expression

**Figure 3:**
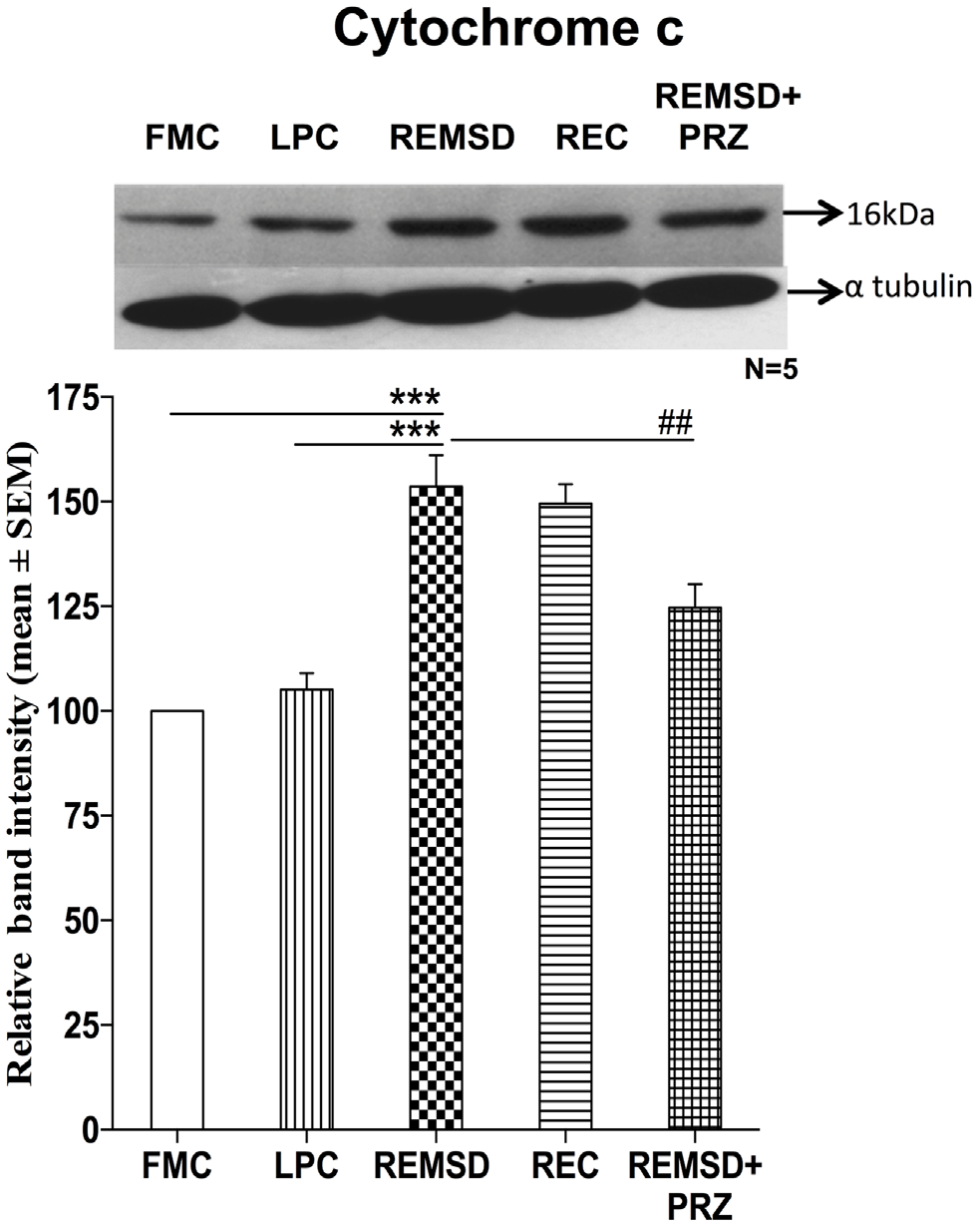
**Cytochrome *c* expression in the brain homogenate of rats under different conditions**. Upper panel shows a representative Western blot of cytochrome *c*. Histogram in the lower panel shows percent changes in the mean (±SEM) band densities of the blots as compared to FMC taken as 100% in five sets of experiments. Abbreviations are as in the text. Significance levels are between the treatments of connecting horizontal bars; ***p < 0.001 and ^##^*p* < 0.01, * as compared to FMC and ^#^ as compared to REMSD.

Apoptosome complex formation starts with the release of cytochrome *c*; it is a crucial step in mitochondria mediated apoptosis. In the present study, we observed that the cytochrome *c* levels were significantly higher in REMSD [*F*_(1,8)_ = 49.64, *p* < 0.001] and REC groups [*F*_(1,8)_ = 115.59, *p* < 0.001] as compared to FMC. The cytochrome *c* levels in LPC rat brains were comparable to that of FMC [*F*_(1,8)_ = 1.64, *p* < 0.23]. PRZ significantly reduced cytochrome *c* levels in the rat brain as compared to REMSD [*F*_(1,8)_ = 9.29, *p* < 0.01]; however, it could not completely prevent the increase, which remained higher as compared to FMC [*F*_(1,8)_ = 19.18, *p* < 0.002] and LPC [*F*_(1,8)_ = 8.03, *p* < 0.02] (Figure 3).

### Apoptosis Protease Activating Factor-1 Expression

**Figure 4:**
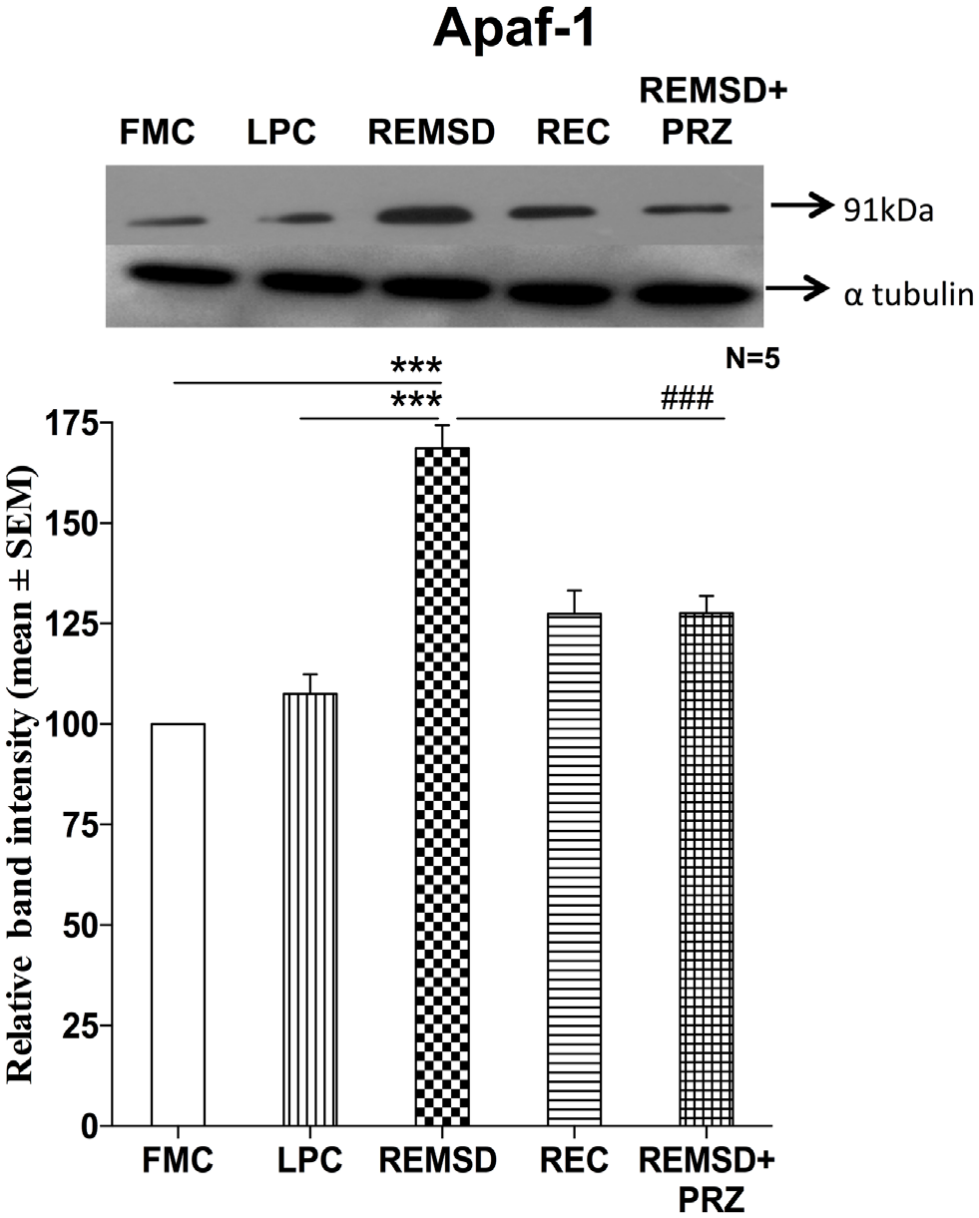
**Apaf-1 expression in the brain homogenate of rats under various conditions**. Upper panel shows a representative Western blot of Apaf-1. Histogram in the lower panel shows percent changes in the mean (±SEM) band densities of the blots as compared to FMC taken as 100% in five sets of experiments. Abbreviations are as in the text. Significance levels are between the treatments of connecting horizontal bars; ***p < 0.001 and ^##^*p* < 0.01, * as compared to FMC and ^#^ as compared to REMSD.

We estimated Apaf-1 in the same brain homogenate used for estimating BAD and cytochrome *c*. Western blot analysis showed significantly higher expression of Apaf-1 in REMSD [*F*_(1,8)_ = 140.51, *p* < 0.001] and REC [*F*_(1,8)_ = 22.19, *p* < 0.002] rat brains as compared to the FMC. The expressions in LPC rats were comparable to that of FMC [*F*_(1,8)_ = 2.35, *p* < 0.16]. As compared to REMSD group, the Apaf-1 expressions decreased in PRZ group [*F*_(1,8)_ = 37.73, *p* < 0.001]; however, the expression continued to remain higher than in the samples from the FMC [*F*_(1,8)_ = 43.19, *p* < 0.001] and LPC [*F*_(1,8)_ = 9.73, *p* < 0.01] (Figure 4).

### Caspase9 Expression

**Figure 5:**
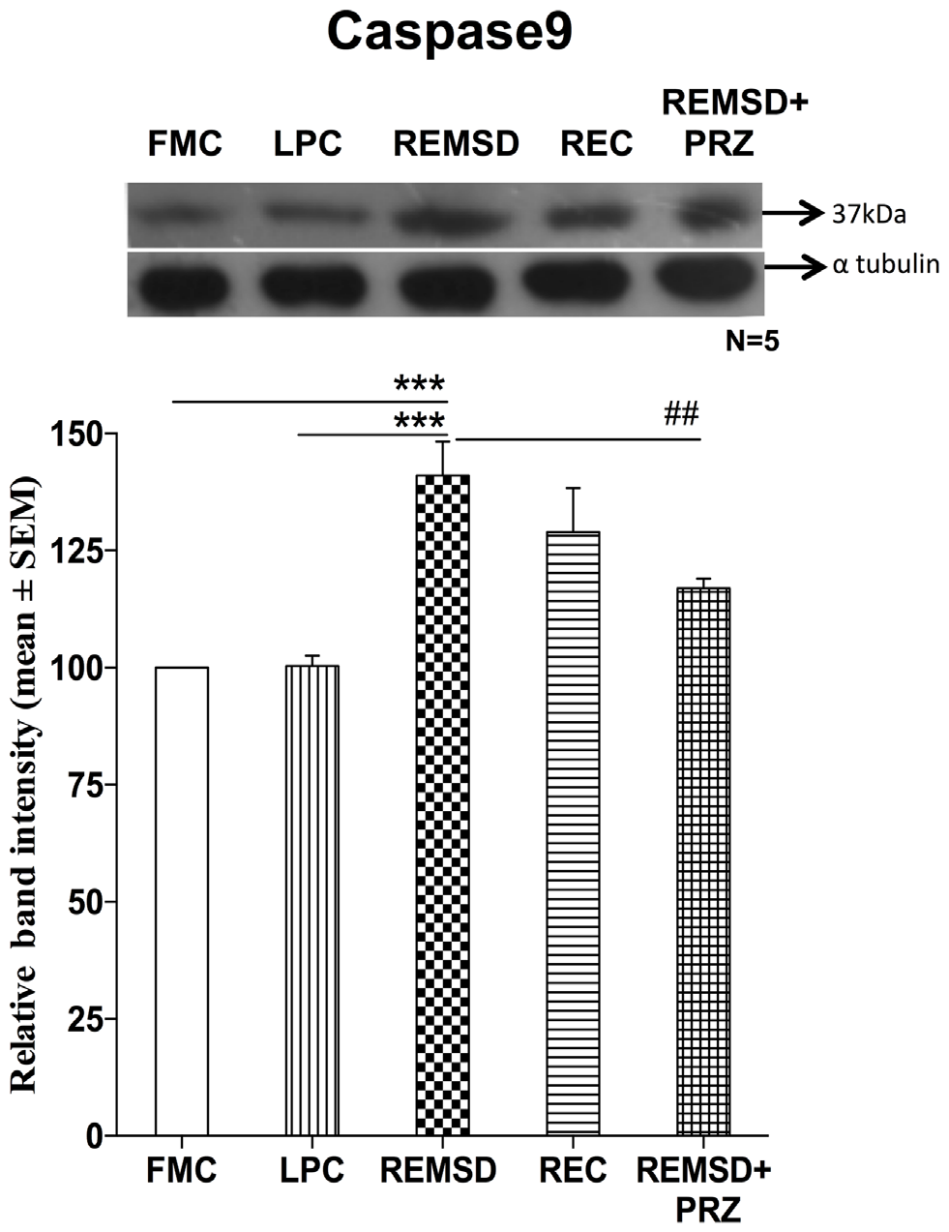
**Caspase9 expression in the brain homogenate of rats under different conditions**. Upper panel shows a representative Western blot of caspase9. Histogram in the lower panel shows percent changes in the mean (±SEM) band densities of the blots as compared to FMC taken as 100% in five sets of experiments. Abbreviations are as in the text. Significance levels are between the treatments of connecting horizontal bars; ***p < 0.001 and ^##^*p* < 0.01, * as compared to FMC and ^#^ as compared to REMSD.

There was a significant increase in the levels of caspase9 expression in REMSD group as compared to FMC [*F*_(1,8)_ = 31.94, *p* < 0.001]; it was unaffected in the LPC [*F*_(1,8)_ = 0.02, *p* < 0.87]. The significant upregulation of caspase9 continued even after 3 days of REC [*F*_(1,8)_ = 9.50, *p* < 0.02]. The REMSD-induced increase in caspase9 was significantly reduced by PRZ treatment [*F*_(1,8)_ = 10.17, *p* < 0.01] as compared to REMSD, although the level continued to remain higher than that of FMC [*F*_(1,8)_ = 65.30, *p* < 0.001] (Figure 5).

### Caspase3 Activation

**Figure 6:**
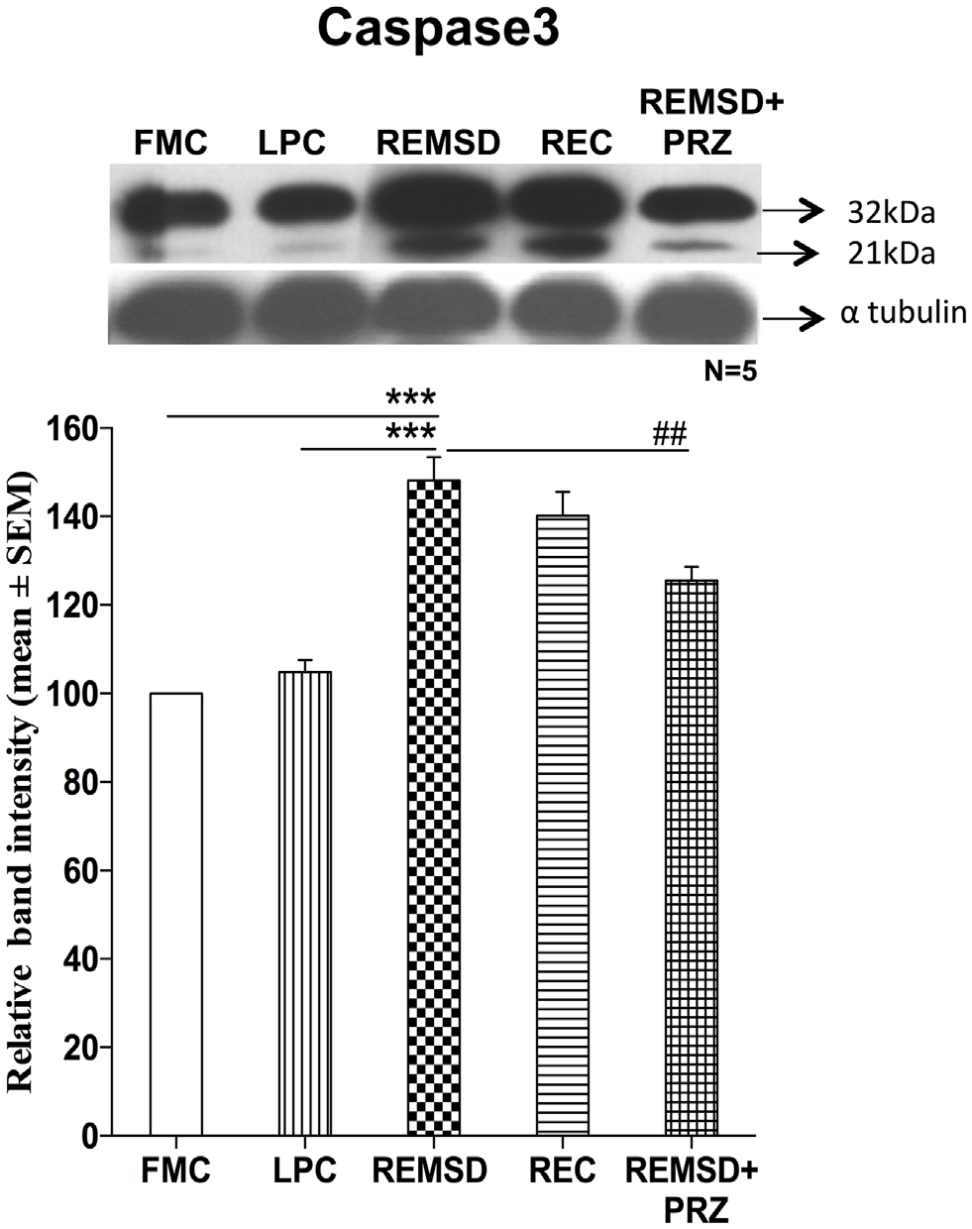
**Caspase3 expressions in the brain homogenate of rats under different conditions**. Upper panel shows a representative Western blot showing the presence of procaspase3 and cleaved caspase3. Histogram in the lower panel shows percent changes in the mean (±SEM) band densities, which were sum of both the Procaspase3 and cleaved caspase3 as compared to FMC taken as 100% in five sets of experiments. Abbreviations are as in the text. Significance levels are between the treatments of connecting horizontal bars; ***p < 0.001, * as compared to FMC and # as compared to REMSD.

The effect of REMSD on caspase3 activation was evaluated by immunoblot analysis of inactive procaspase3 (32 kDa) and activated form of caspase3 (21 kDa). As compared to FMC, the REMSD significantly elevated their levels [*F*_(1,8)_ = 82.45, *p* < 0.001] and the increase continued even after REC [*F*_(1,8)_ = 55.44, *p* < 0.001]. In LPC rats, the levels remained statistically comparable with FMC [*F*_(1,8)_ = 3.01, *p* < 0.12]. PRZ prevented the activation of caspase3 as compared to REMSD alone [*F*_(1,8)_ = 13.50, *p* < 0.006]. Band intensities of both procaspase3 (32 kDa) and cleaved Caspase3 (21 kDa) were taken together for evaluation (Figure 6).

### Phosphorylated Akt Levels

**Figure 7:**
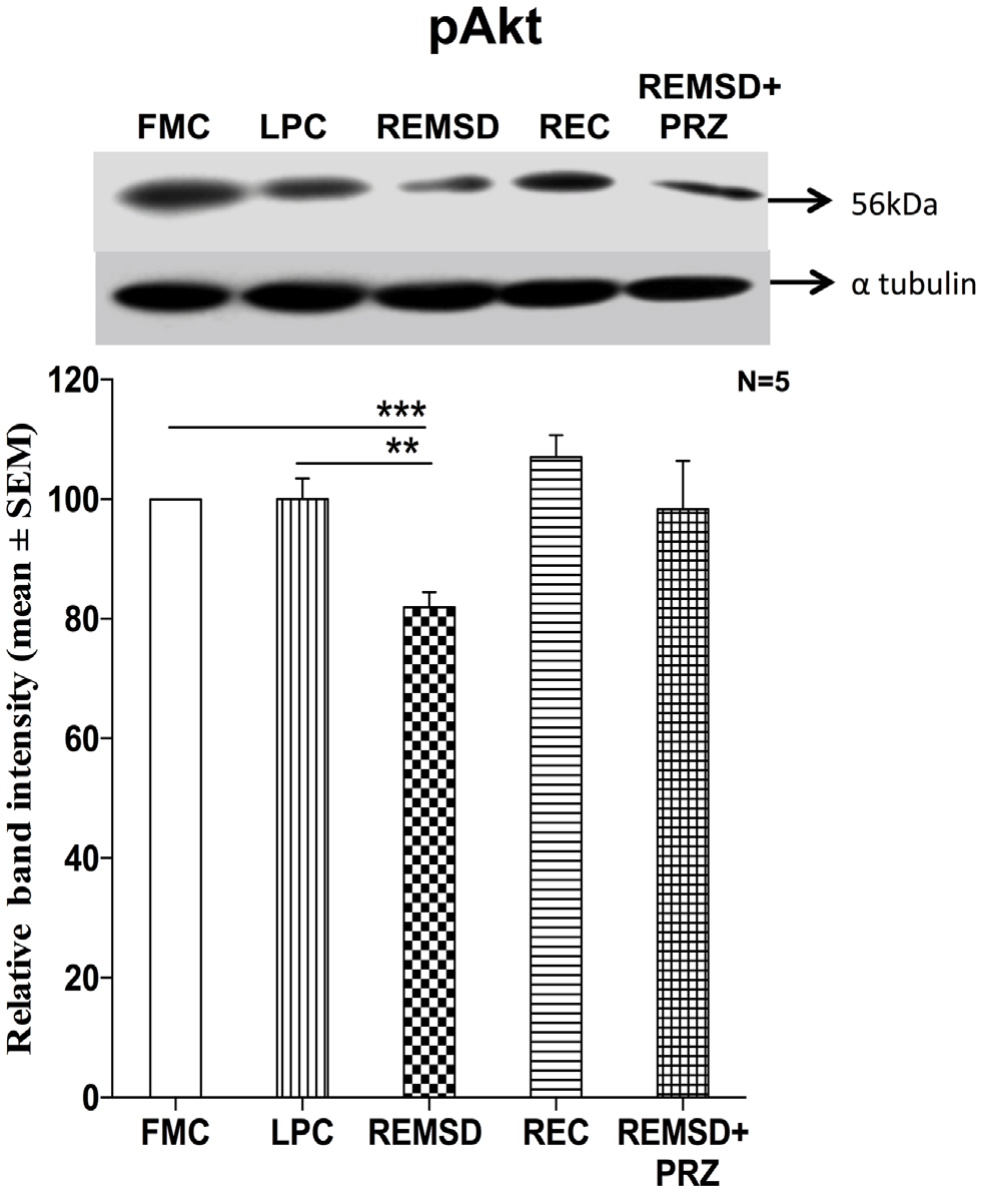
**pAkt expression in the brain homogenate of rats maintained under various conditions**. Upper panel shows a representative Western blot of pAkt. Histogram in the lower panel shows percent changes in the mean (±SEM) band densities of the blots as compared to FMC taken as 100% in five sets of experiments. Abbreviations are as in the text. Significance levels are between the treatments of connecting horizontal bars; ***p < 0.001 and ^#^*p* < 0.05, * as compared to FMC and ^#^ as compared to REMSD.

We estimated the amount of Ser473 pAkt in the control and REMS-deprived rat brains as a measure of activation of the PI3-Kinase/Akt pathway. pAkt levels were reduced significantly in the brain sample of REMSD group as compared to FMC [*F*_(1,8)_ = 49.67, *p* < 0.001]; its levels in LPC were comparable to that of FMC [*F*_(1,8)_ = 0.01, *p* < 0.99]. The reduction in pAkT levels was prevented by PRZ and the levels were comparable to that of FMC [*F*_(1,8)_ = 3.76, *p* < 0.08]. After 3 days of recovery (REC group), the pAkT levels returned to a level comparable to FMC [*F*_(1,8)_ = 3.75, *p* < 0.08] (Figure 7).

### Caspase8 Expression

**Figure 8:**
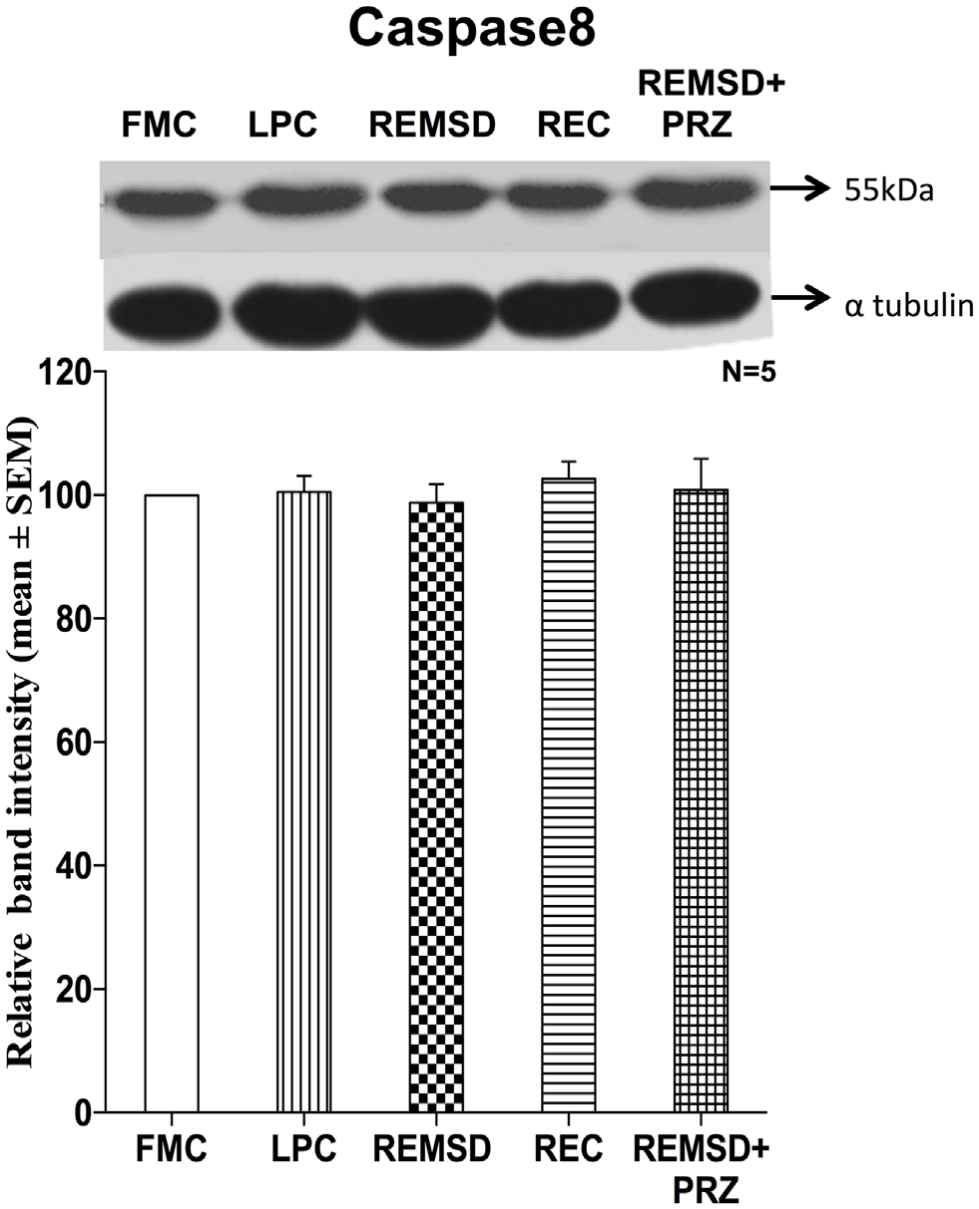
**Caspase8 expression in the brain homogenate of rats maintained under different conditions**. Upper panel shows a representative Western blot of caspase8. Histogram in the lower panel shows percent changes in the mean (±SEM) band densities of the blots as compared to FMC taken as 100% in five sets of experiments. Abbreviations are as in the text.

To evaluate if extrinsic pathway could be involved in the apoptotic process, we estimated the level of caspase8 in the brain samples of REMSD and control rats. Six-day REMSD did not affect the caspase8 expression as compared to FMC [*F*_(1,8)_ = 0.16, *p* < 0.69]. It is evident from the data that the levels remained unaffected in LPC [*F*_(1,8)_ = 0.03, *p* < 0.86], REC [*F*_(1,8)_ = 0.91, *p* < 0.36], and PRZ [*F*_(1,8)_ = 0.02, *p* < 0.87] treated groups as compared to the FMC (Figure 8).

### Tyrosine Hydroxylase Expression

**Figure 9:**
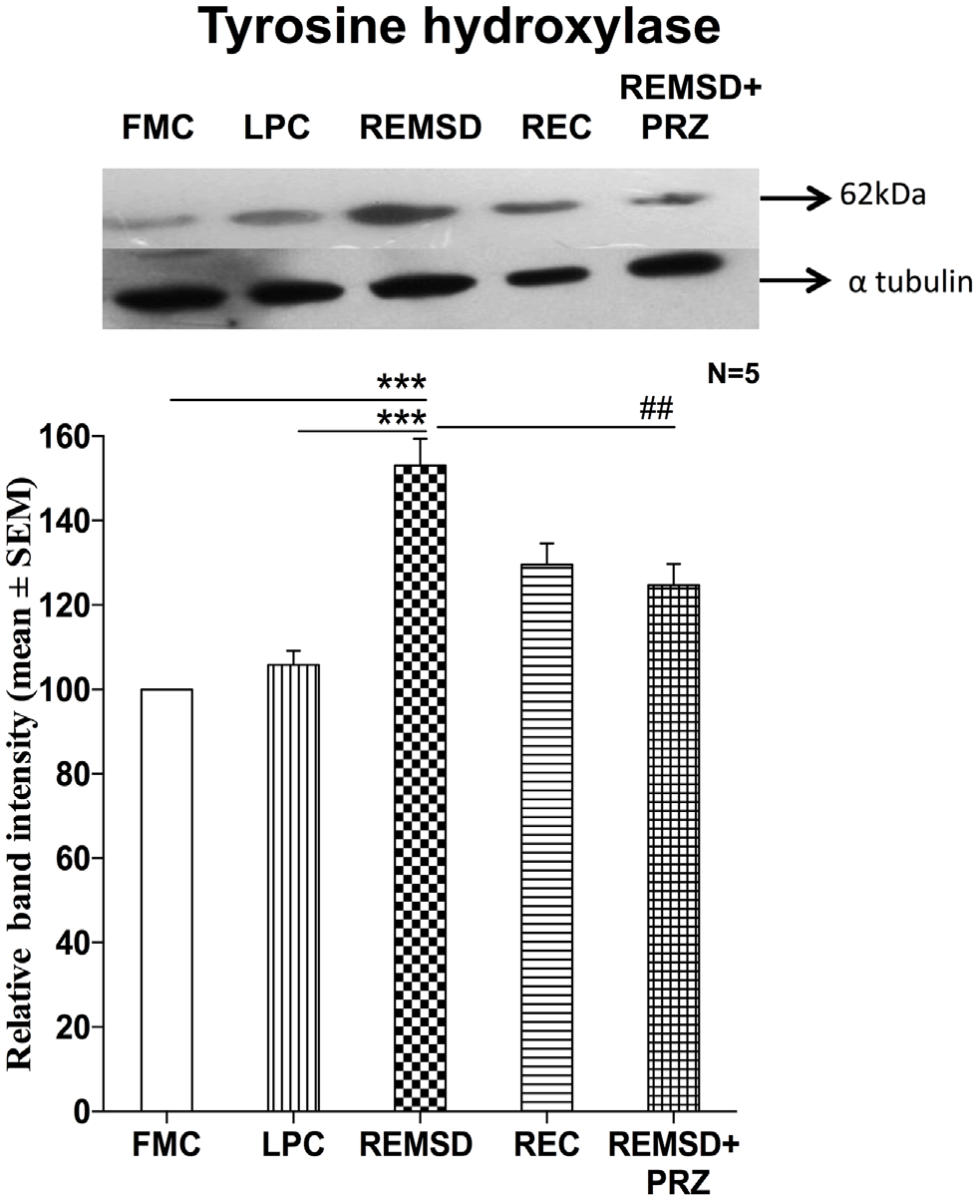
**Tyrosine hydroxylase expression in the brain homogenate of rats maintained under different conditions**. Upper panel shows a representative Western blot of tyrosine hydroxylase. Histogram in the lower panel shows percent changes in the mean (±SEM) band densities of the blots as compared to FMC taken as 100% in five sets of experiments. Abbreviations are as in the text. Significance levels are between the treatments of connecting horizontal bars; ***p < 0.001 and ^#^*p* < 0.05, * as compared to FMC and ^#^ as compared to REMSD.

As the effects described above were prevented by PRZ, an alpha1 adrenoceptor antagonist, it was obvious that NA was involved in the process. Further, as the effects were observed after several days of REMSD, we argued that the synthesis machinery of NA was likely to have been upregulated inducing the effects. To confirm, we estimated level in control and REMSD rat brain samples as TH is the crucial first rate limiting step for the synthesis of NA. We observed that the TH expression was significantly higher in the REMSD rat brains as compared to FMC [*F*_(1,8)_ = 68.69, *p* < 0.001] and REC [*F*_(1,8)_ = 34.59, *p* < 0.001]. The expression levels in LPC were statistically comparable to that of FMC [*F*_(1,8)_ = 2.92, *p* < 0.12]. PRZ [*F*_(1,8)_ = 12.09, *p* < 0.008] prevented the REMSD-associated increase in TH expression (Figure 9) suggesting the effects were mediated by NA.

### TH-siRNA in LC-Downregulated TH Expression

**Figure 10:**
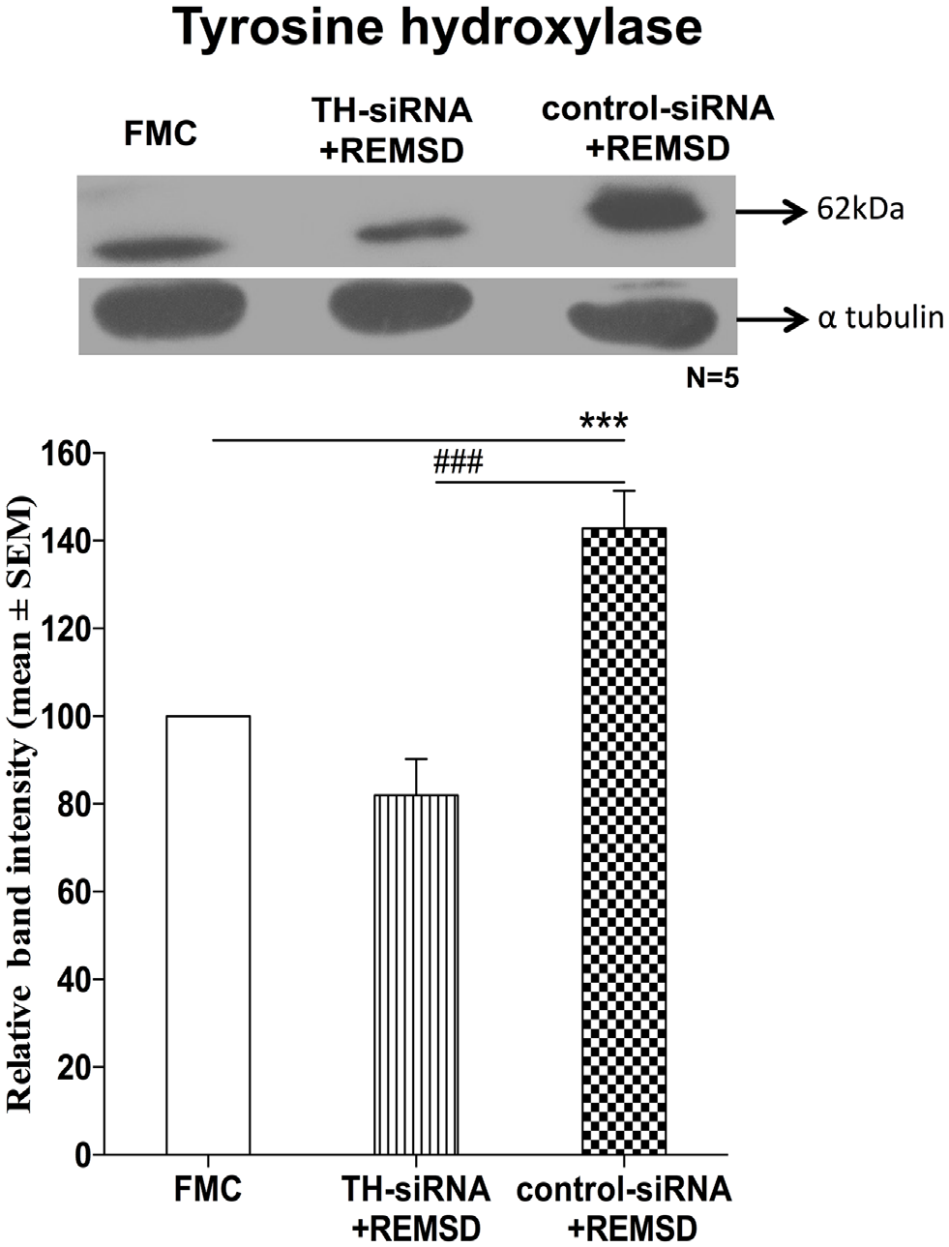
**Expressions of TH in homogenate of REMSD rat brains after microinjection of TH-siRNA or control siRNA bilaterally into LC**. Upper panel shows a representative Western blot of tyrosine hydroxylase. Histogram in the lower panel shows percent changes in the mean (±SEM) band densities of the blots as compared to FMC (without injection) taken as 100% in five sets of experiments. Abbreviations are as in the text. Significance levels are between the treatments of connecting horizontal bars; ***p < 0.001 and ^###^*p* < 0.001, * as compared to FMC and ^#^ as compared to TH-siRNA + REMSD.

Once it was evident that NA synthesis was upregulated upon REMSD, the obvious follow-up question was about the source of NA. The LC is the primary site for NA-ergic neurons in the brain (23), those neurons project throughout the brain (24), they (the REM-OFF neurons) normally cease firing during REMS (25, 26) and they do not cease firing during REMSD (27). Therefore, in separate groups, bilateral LC was infused with either TH-siRNA or control siRNA and the rats were subjected to REMSD. It was observed that as compared to FMC, upon REMSD, TH expressions were upregulated in the brains of rats, which received control–TH-siRNA into the LC [*F*_(1,8)_ = 25.18, *p* < 0.001]; it was not upregulated in the brains of rats, which received TH-siRNA; this supported our views (Figure 10).

### TH-siRNA in LC Suppressed Release of Cytochrome *c*

**Figure 11:**
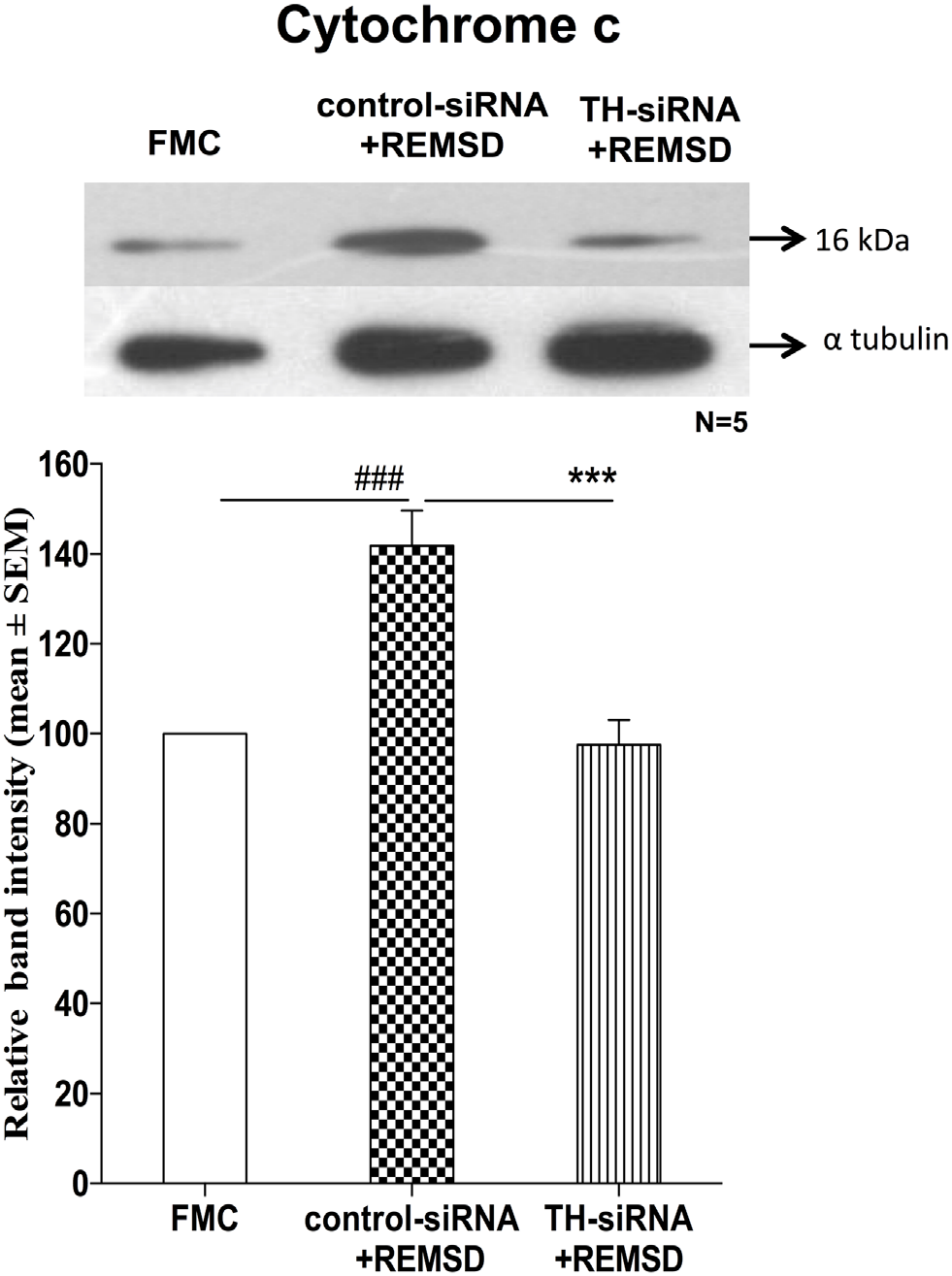
**Expressions of cytochrome *c* in homogenate REMSD rat brain after microinjection of TH-siRNA or control siRNA bilaterally into LC**. Upper panel shows a representative Western blot of cytochrome *c*. Histogram in the lower panel shows percent changes in the mean (±SEM) band densities of the blots as compared to FMC taken as 100% in five sets of experiments. Abbreviations are as in the text. Significance levels are between the treatments of connecting horizontal bars; significance levels – ***p < 0.001 and ^###^*p* < 0.001, * as compared to TH-siRNA and ^#^ as compared to FMC.

We have seen above that REMSD-associated elevated NA induced the release of cytochrome *c*. This suggested activation of mitochondrial intrinsic pathway led to apoptosis. To confirm, we hypothesized that downregulation of NA synthesis by infusing TH-siRNA into the LC, the major source of NA in the brain and then subjecting the rats to REMSD, should not release cytochrome *c*. Therefore, in separate groups of chronically prepared rats, either control siRNA or TH-siRNA (to silence the expression of TH synthesis in the LC neurons) was microinjected bilaterally into the LC *in vivo* and deprived them of REMS. We observed that cytochrome *c* levels were significantly lower [*F*_(1,8)_ = 114.66, *p* < 0.001] in the brains of REMS-deprived rats, which received TH-siRNA as compared to the rats deprived of REMS with control siRNA. The cytochrome *c* levels in the former group of rats (i.e., those received TH-siRNA and REMS deprived) was comparable to its level in the FMC [*F*_(1,8)_ = 1.06, *p* < 0.33]. This indicates that the REMSD-induced elevated release of cytochrome *c* was mediated by NA released from the LC neurons, which was prevented if the synthesis of NA in LC neurons was reduced by downregulating the TH synthesis (Figure 11).

### TEM Evaluation of Morphological Changes in Mitochondria and Nucleus in Neurons after REMSD

**Figure 12:**
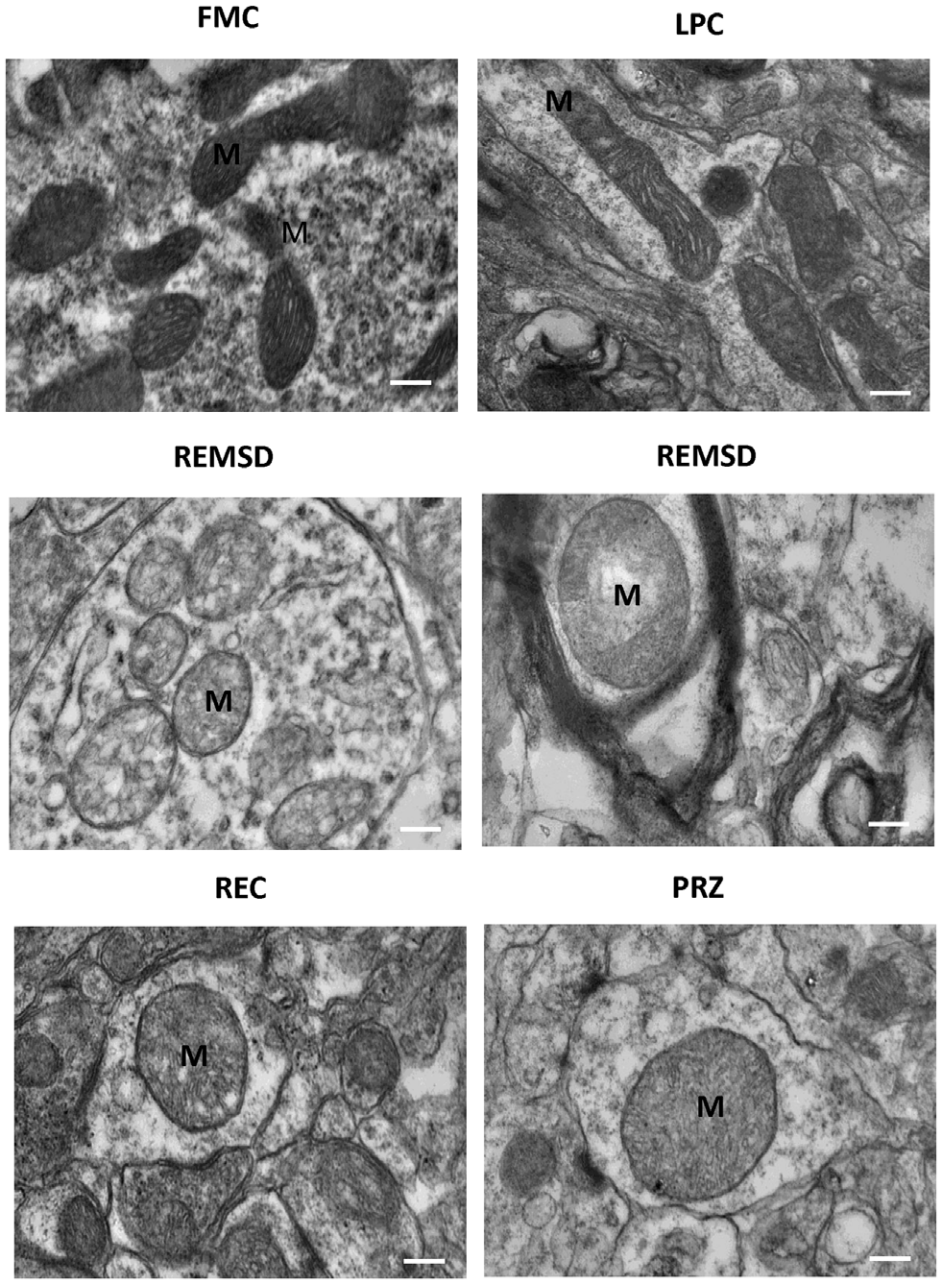
**Electron photo-micrograph (10,000×) showing mitochondrial ultrastructural changes in LC neurons of rats treated under various conditions**. Normal elongated mitochondria can be seen in neurons from FMC and LPC rat brains. Images of two different rat brains after REMSD are shown as compared to one each from other control rat brains. Mitochondria appeared swollen with cristae disintegrated or reduced with transparent matrix in the REMSD rat brains. Cristae appeared less distorted in REC rat brain. In PRZ-treated group, although swelling appeared, the inner cristae of mitochondria appeared intact. M – mitochondria; scale bar: 100 nm.

**Figure 13:**
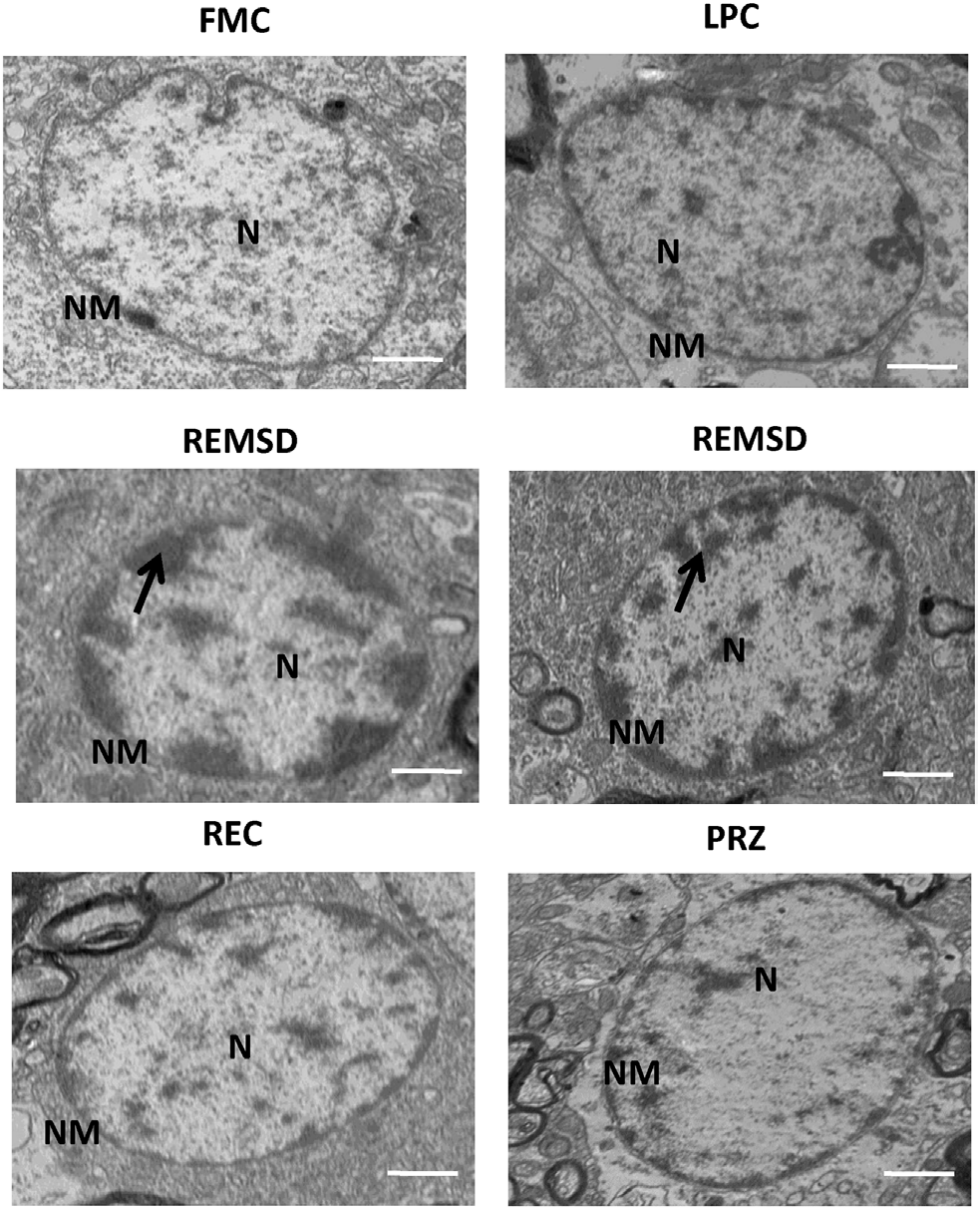
**Electron photo-micrograph (2,500×) of nucleus in LC neurons of rat brains treated under different conditions**. Nucleus and chromosomes appear normal in FMC and LPC. Images of two different rat brains after REMSD are shown as compared to one each from other control rat brains. Neuron with chromatin condensation can be seen in the REMSD rat brains. The dark arrows point to areas near nuclear membrane with increased chromatin condensation. Reduced degenerative changes of nucleus were seen in the REC- and PRZ-treated rat brains. N – nucleus, NM – nuclear membrane; scale bar: 500 nm.

Rod-shaped mitochondria with intact cristae and membrane were seen in the neurons in FMC and LPC groups of rats; however, they appeared distinctly swollen upon REMSD. The cristae appeared disintegrated and significantly reduced with transparent matrix in the REMSD rat neurons, whereas the cristae were less distorted in REC group of rats. The integrity of mitochondrial cristae in the neurons of PRZ-treated REMS-deprived rats appeared similar to those of FMC and LPC rats (Figure 12). The chromosomal DNA within the nuclei of neurons in the REMS-deprived rats appeared condensed and clumped along the nuclear membrane suggesting increased chromosomal degradation and apoptosis. Fewer neurons (than REMSD group) in REC and PRZ-treated groups showed such signs of apoptosis (Figure 13). This suggested that REMSD-associated apoptosis and neurodegeneration were induced by NA acting on alpha1 adrenoceptors. However, the effect was reversed if the NA level was naturally reduced in REC rats.

## Discussion

Based on consistent and substantial evidences, it has been proposed that REMS serves house-keeping function of the brain (9). It is affected in almost all psychosomatic disorders (28, 29) and neurodegenerative diseases (30–33). Prolonged REMS disturbance has been proposed to be a significant cause, facilitator, or pre-disposing factor of neurodegeneration (34). We have shown earlier that upon REMSD, there was significant reduction in cytoskeletal proteins, changes in neuronal cytomorphology, and in the levels of pro- and anti-apoptotic proteins, respectively, in the rat brain (2); however, the detailed mechanism(s) of inducing such changes were unknown. Notwithstanding, it has been reported that cessation of the LC-NA-ergic REM-OFF neurons is a prerequisite for the generation of REMS (35) and if those neurons are kept active, REMS does not appear (36). Because of the latter, i.e., continuous activity of the NA-ergic neurons, the levels of NA increase in the brain upon REMSD and that induces many of the REMS loss-associated effects and symptoms from gene to molecule to behavior (37). Therefore, we proposed that elevated levels of NA could be at least a major causative factor for REMSD-associated neuronal apoptosis in the brain. Accordingly, we have studied the molecular mechanism of REMSD-associated NA-mediated apoptosis in the rat brain and if elevated NA induced such effects. We observed that in the rat brain, REMSD-induced apoptosis occurs by the activation of the mitochondrial intrinsic pathway and that is initiated by elevated level of NA acting on alpha1 adrenoreceptor in the brain.

The mitochondrial intrinsic pathway-induced apoptosis in mammals is regulated by Bcl2 family of proteins upstream of caspase activation (38). The Bcl2 family of proteins has either pro- (e.g., BAX, BAD) or anti- (e.g., Bcl2, Bcl-xl) apoptotic property that regulates permeabilization of the outer mitochondrial membrane. In healthy cells, the proapoptotic factor BAD is phosphorylated by Akt and sequestered into the cytoplasm by the tau form of 14-3-3 protein and precludes its binding to Bcl2, thereby enhancing survival pathway (39). In this study, we have observed that upon REMSD, the levels of the survival protein pAkt were significantly reduced, while the un-phosphorylated BAD levels were significantly elevated in the rat brain; the latter is likely to be due to reduced levels of pAkt. BAD promotes cell death by binding to anti-apoptotic members of the Bcl2 family and thus inhibiting their survival promoting functions (40). Normally, Bcl2 interacts with BAX to form a complex that prevents apoptosis, while BAD breaks the complex releasing BAX into the cytosol favoring apoptosis (41). Earlier we have shown that BAX level is elevated in the brain under identical REMSD condition (2). Thus, it appears that the BAX released from the Bcl2 is translocated into the mitochondria and favors apoptosis, possibly by the release of cytochrome *c*.

To confirm, we estimated the relative expression of cytochrome *c* in experimental (REMSD) and control rat brains and found that cytochrome *c* levels were higher in REMS-deprived rat brain as compared to the controls. It is known that the released cytochrome *c* promotes multimerization of Apaf-1 to form apoptosome complex, which sequentially recruits and activates initiator caspase9 (42) that elicits downstream caspase cascade (43). In the present study, we found that Apaf-1 and caspase9 levels were elevated in REMSD rat brain suggesting that upon REMSD, the elevated Apaf-1 and cytochrome *c* were likely to be involved in apoptosome formation resulting in activation of caspase9. The activation of caspase9 is known to further cleave and activate caspase3, thereby setting in motion the events that led to DNA fragmentation and cell death (44). We observed that upon REMSD, there was significantly higher level of caspase3 activation, a hallmark of apoptosis. The activation of caspase3 may take place by following two main pathways. One, the extrinsic route initiated by activation of the cell surface receptors leading directly to caspase8 activation, and two, the intrinsic pathway that is regulated by mitochondria (45); findings of this study support that the latter was active.

In an attempt to understand the mechanism of REMSD-induced increased apoptosis, we targeted NA as it is one of the key neurotransmitters regulating REMS. NA is elevated during REMSD, and it is responsible for many REMSD-associated cellular, molecular, and psychosomatic behavioral changes (9, 46). Our hypothesis was indeed supported by the fact that the levels of all the proapoptotic proteins viz. BAD, Apaf-1, cytochrome *c*, and caspase9 were significantly reduced in PRZ-treated REMSD rat brains as compared to that of the untreated but deprived rat brains. As PRZ is an α1-adrenoceptor antagonist, the results suggest that the expressions of the proapoptotic proteins in the REMSD rat brains were induced by REMSD-associated elevated level of NA acting on α1-adrenoreceptors in the rat brains. However, one may argue that such decreased levels of the apoptotic factors were still statistically not comparable to their respective levels of proteins in the FMC and LPC rat brains. This could be because either or combination of the following factors. Several days of REMSD might have affected other physiological processes to induce changes in the apoptotic factors, higher dose of PRZ possibly was required, other adrenoceptors and factors were involved in mediating the effects, and other pathways could be involved in the process and possible complex crosstalks among several factors and molecules for inducing these changes.

To re-confirm further that increased apoptosis was due to REMSD-associated elevated levels of NA in the brain and in an attempt to simulate the condition *in vivo*, we hypothesized that manipulation of the brain NA-ergic system, which is critically involved in REMS regulation would induce the REMSD-associated changes. As it was known that cessation of NA-ergic LC neurons is a prerequisite for REMS regulation, those neurons do not cease activity during REMSD; NA from those neurons induces several other REMSD-associated effects, and NA-synthesizing enzyme (i.e., TH) is increased upon REMSD (9, 46, 47); we hypothesized that if NA synthesis was downregulated, REMSD should not be able to express the REMSD-associated effects in spite of behavioral REMSD. Accordingly, while depriving, the NA synthesis from LC neurons was downregulated *in vivo* by local microinjection of TH-siRNA bilaterally into the rat LC. As TH levels were significantly reduced in the brains of these rats, it may be inferred with reasonable certainty that NA levels were significantly reduced in them. Further, indeed in these rats, cytochrome *c* levels did not increase even after REMSD although cytochrome *c* levels remained elevated in the REMS-deprived rats that received control siRNA in the LC. It was technically not possible to confirm the microinjection site and at the same time use the brain homogenate for estimation of other proteins from the same brain sample. However, using reductionist approach of findings from various control as well as pilot experiments, it can be said with reasonable confidence that the microinjections were in the LC. For example, if the microinjections were not in the LC, reduction in TH in LC-injected rats, but not in control rats (Figure 10), cannot be justified. All these findings taken together suggest that upon REMSD, the NA level increases in the brain and the elevated level of NA induces cytochrome *c* level, which in turn induces neuronal apoptosis in the brain. Further, REMSD-induced apoptosis followed mitochondrial intrinsic pathway as caspase9, caspase3, and cytochrome *c* were elevated, while caspase8 remained unaffected. Since changes in cytochrome *c* levels play a pivotal role in the mitochondrial intrinsic pathway, we estimated its levels in TH-siRNA-injected REMS-deprived rat brains. As critical argument, it may be emphasized that TH silencing at least inhibits initiation of intrinsic apoptotic pathway in REMS-deprived rats in particular.

In many chronic neuropathological conditions, the REMS is reported to decrease with associated loss of LC neurons (34, 48). The latter effect is likely to reduce NA in the LC projection areas in such chronic condition; however, it would depend on the proportion of NA-ergic cell loss, their recovery, efficiency of NA synthesis, and so on. Our views may be supported by the fact that no change, significantly reduced or elevated levels, of NA in the brain and cerebrospinal fluid of the Alzheimer’s patients have been reported (48–50). We argue that in most such neuropathological condition, if not all, at least initially (at the onset), the REMS is decreased raising the level of NA in the brain. If the elevated level of NA is sustained without compensation (control), neuronal apoptotic process sets-in giving rise to varieties of symptoms, which depend on sensitivity, predisposition, brain area, number of neurons damaged, and so on. Notwithstanding, it is also possible that the NA level might increase in the brain for various reasons including life style and other diseases causing reduced REMS, which then reinforces elevated levels of NA leading to apoptosis. We have dealt with neural mechanism of REMS regulation and elevation of NA upon REMSD at length elsewhere (9, 46, 47). Because of the above, naturally the symptoms vary, although reduced REMS remains a common associated symptom in neurodegenerative diseases and level of NA would depend on the chronicity of the case, how many LC neurons are effectively damaged, how much NA synthesis is affected, and so on by the time the patients reach the clinics. Thus, although desirable, it may be difficult to track the sequence of cause and effect events in patients; however, reduced REMS and elevated level of NA cannot be denied, which then initiates apoptosis; its molecular mechanism has been traced in this study.

Rats were REMS deprived by the flower pot method, the most preferred method for inducing experimental REMSD. Earlier we have discussed in detail the advantages and disadvantages of this method (51), where the former outweighs the latter. By this method, non-REMS is also significantly reduced if the deprivation is continued for 24 and 48 h; however, REMS is maximally reduced and non-REMS is least affected (comparable to that of LPC rats), if the deprivation is continued for longer period as has been done in this study. Notwithstanding, a common criticism of most behavioral studies is induction of possible stress to the subjects caused by the experimental manipulation, and this study too cannot escape from the same criticism. The rats undergoing REMSD may experience stress due to restricted movement, social isolation, and muscular overactivity. To rule out the effects of such non-specific factors confounding the results, we have used LPC and REC. Earlier, using the same method, we have shown that at least for Na-K ATPase activity, the effects were due to REMSD and not due to other non-specific factors (52). Although stress has been defined and characterized in more than one way, some studies have correlated stress to elevated levels of NA and corticosterone (53, 54). REMSD-associated elevation of NA level can be explained by the fact that the NA-ergic REM-OFF neurons, which normally cease activity during REMS, continue activity during REMSD (27, 46). Therefore, in this study, we have investigated the effect of NA on neuronal apoptosis. As the REMSD-associated effects were prevented by NA-ergic antagonist, PRZ and by downregulation of NA synthesis in the LC neurons, it is reasonable to infer that the observed effects were due to the elevated levels of NA acting on alpha1 adrenoceptors. The effect of PRZ *per se* may be ruled out as PRZ treatment is reported to induce TH expression (55) rather than decrease, as has been observed in this study. As corticosterone is neither directly involved in the regulation of REMS nor (more importantly) its level increases upon REMSD even using the method we have used (5, 37, 56, 57), it is highly improbable that the effects observed in this study were induced by corticosterone.

The levels of proapoptotic proteins in the LPC rat brains were comparable to respective FMC levels, suggesting that the changes observed in the experimental REMS-deprived rat brains were not due to non-specific factors. Further, the observed effects were specific to REMSD because most of the induced effects (e.g., pAkt) either returned or tended to return toward the baseline (FMC) level after the rats were allowed to recover (REC) from the lost REMS. The effects were not induced by non-specific factors because some of the proteins remained unaffected (e.g., caspase8) after REMSD; all the effects were not affected uniformly and some of the changes (e.g., BAD, cytochrome *c*, Apaf-1, caspase9, and caspase3) did not return completely to the FMC (basal) level in REC rats. The latter could be because the threshold of recovery time may differ for different proteins (factors) and possibly more time was necessary for recovery.

Rapid eye movement sleep deprivation affected the molecules under study in the whole brain homogenate. This suggested that REMSD exerts a generalized effect in most neurons throughout the brain and it explains why REMSD affects most of the physiological processes globally in the body particularly controlled by the brain. However, the intensity of effects differs possibly due to sensitivity and predisposition of the affected neurons as well as the physiological processes. These views may be supported by the fact that LC projects to all parts of the brain (58), and we have reported that the effects of REMSD start from localized region and then spread to different regions in the brain with increase in duration of REMSD (59). At the molecular level, it can be further attributed that executioner caspase (e.g., caspase3) is autocatalytically activated and once the level of the mitochondrial apoptotic signaling reached a critical value, “*a set-point* or *a point of no-return*,” the effect of anti-apoptotic Bcl-2 protein escapes exerting protecting effect; however, it does not follow all and none law. Furthermore, interestingly, as most of the changes returned or tended to return upon recovery of REMS (e.g., pAkt), it reflects that apoptosis is not an irreversible process and quite logically there are self-rectifying physiological processes, which when fails (*point of no-return*), apoptotic damage is initiated.

Perturbations in mitochondrial respiration can occur early in the apoptotic process; mitochondria itself may serve as a control switch at least for some form of apoptosis (60). Mitochondrial shape, size, and numbers vary within neurons and synapses. To substantiate our findings, we carried out TEM studies to qualitatively evaluate the physical damage to the mitochondria, the shape, and intactness (breakage) of cristae, in particular. Our TEM results showed physical damage in the mitochondrial ultrastructure in REMS-deprived rats. Swelling of the mitochondrial matrix was observed in the LC neurons upon REMSD. Such mitochondrial changes have been reported to be the immediate cause of cytochrome *c* release (61), which further supports our findings of elevated cytochrome *c* level in the brain after REMSD. Mitochondrial impairment has been shown to promote apoptotic cell death with depolarization associated with release of apoptosis-inducing factor and cytochrome *c*, and activation of procaspases (62). Recently, reduced levels of SirT3 have been correlated to reduced number of neurons in mice kept awake (63, 64). As increased waking is associated with REMSD, whether the effects seen in this study could have any association/relation with Sirt3 levels need further study.

The ultrastructural changes in the mitochondria were induced by REMSD-associated elevated level of NA in the brain because the effects were reduced by PRZ, and it showed partial recovery with post-REMSD recovery of REMS, when the level of NA returned to normal level. Although apparently, there might be minor differences in the size of mitochondria in the FMC and LPC rat brains, they were generally elongated (not round or oval) and cristae were mostly intact. A complete reversal of the mitochondrial physical characteristics to normal elongated shape was not seen after 3 days of REC. This is possibly because more time was needed for complete recovery, which may be supported by the fact that cytochrome *c* levels remained elevated in REC- and PRZ-treated rat brains as compared to FMC and LPC. The morphological changes in the mitochondria support that REMSD-associated apoptotic changes (2) follow mitochondrial intrinsic pathway and the effects were mediated by NA acting on alpha1 adrenoreceptor.

**Figure 14:**
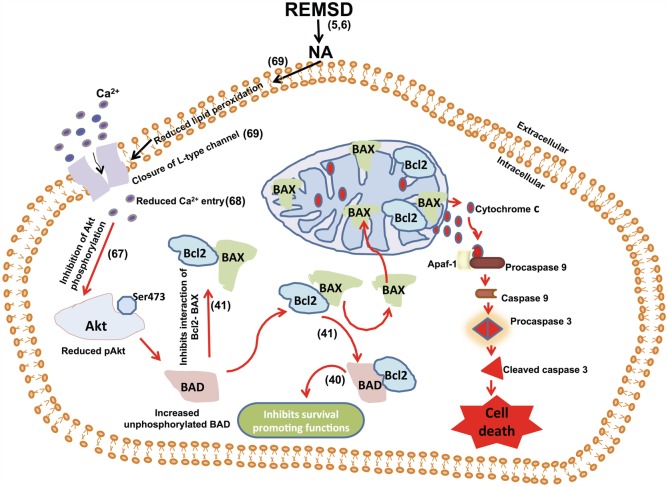
**We have shown earlier that REMSD increases apoptosis in the rat brain and that is mediated by REMSD-associated elevated NA level in the brain (1)**. We have also shown that REMSD reduces intracellular Ca^2+^ (68) and NA reduces Ca^2+^ influx (69). Combining those findings with the results of this study that the REMSD-associated neuronal apoptosis follows mitochondrial intrinsic pathway, we have traced the molecular pathway, which has been schematically represented in this figure.

Reduction in pAkt by NA upon REMSD may be justified by the fact that Akt has been reported to promote cell survival (65, 66). Although we have not studied its intracellular mechanism of action directly in this study, based on our previous reports under similar condition and supported by other findings, it may be explained as follows. Ca^2+^ plays a role in phosphorylation of Akt for its activation (67). We have reported reduction in intra-synaptosomal Ca^2+^ level under similar REMSD condition (68) and NA reduces Ca^2+^ influx (69). Therefore, combining our previous observations and findings of this study, we conclude that REMSD elevates NA, which in turn reduces Ca^2+^ influx resulting in reduced phosphorylation of Akt. The latter activates BAD triggering mitochondrial intrinsic pathway resulting neuronal apoptosis, while BAD combining with Bcl2 inhibits neuronal survival (Figure 14). We also propose that intensity and severity of REMSD would depend on synergism of effects and predisposition of the affected neurons to induce pathophysiological changes. If the catastrophic cascade at the molecular level would reach a *point of no-return* (set-point), neurodegeneration would be initiated. However, the silver lining is that the effects may be prevented by compensating lost REMS and/or by reducing NA synthesis by possible therapeutic intervention, which we have shown as a proof of principle.

## Author Contributions

BM designed the experiment. BS performed experiment and analyzed the data. MK performed stereotaxic surgery and microinjections. All authors have contributed to the final preparation of the manuscript.

## Conflict of Interest Statement

The authors declare that the research was conducted in the absence of any commercial or financial relationships that could be construed as a potential conflict of interest.

## Abbreviations

Apaf-1, apoptosis protease activating factor-1; BAD, Bcl-2-associated death promoter; BAX, Bcl-2-associated X protein; Bcl2, B-cell lymphoma 2; LC, locus coeruleus; NREMS, non-REMS; pAkt, phosphorylated Akt (Ser 478); REMS, rapid eye movement sleep; REMSD, REMS deprivation; siRNA, small interfering RNA; TH, tyrosine hydroxylase.
